# Liver enzyme levels are associated with markers of systemic inflammation, disease severity, and metabolic syndrome in patients with hidradenitis suppurativa

**DOI:** 10.1002/hsr2.360

**Published:** 2021-09-16

**Authors:** Valdemar Wendelboe Nielsen, Jesper Grønlund Holm, Astrid‐Helene Ravn Jørgensen, Yiqiu Yao, Hans Christian Ring, Simon Francis Thomsen

**Affiliations:** ^1^ Department of Dermato‐Venereology & Wound Healing Centre Bispebjerg Hospital Copenhagen Denmark; ^2^ Department of Biomedical Sciences University of Copenhagen Copenhagen Denmark

## 1 INTRODUCTION

In the understanding of hidradenitis suppurativa (HS) as a follicular occlusion disorder with increased systemic inflammatory load, comorbidities across multiple organ systems have been gradually recognized. Recently, metabolic syndrome (MetS) has been linked with HS.^1^ The aims of this study were to evaluate liver function in patients with HS, and the potential association between liver enzymes and systemic inflammatory load, disease severity, and presence of MetS.^2^, ^3^

## 2 METHODS

A total of 470 outpatients from a university department from January 2016 to May 2020 were included (Table 1). Information on age, sex, smoking, ethnicity, body mass index (BMI), disease duration, and number of boils in the last month was noted. Furthermore, the presence of systemic comorbidities and severity of HS were obtained through clinical examination and confirmed from diagnoses in patient files. All patients with HS referred to the department were eligible to participate in the study, after written consent was obtained.

**TABLE 1:** Demographic factors and disease characteristics of patients with hidradenitis suppurativa

	Total, n = 470	Female, n = 301 (64%)	Male, n = 169 (36%)	*P*‐value
Demographic factors				
Age [mean (range)] (years)	40 (10‐92)	38 (13‐92)	43 (10‐73)	<.001
Smoking status, n (%)				.01
Present smoker	262 (55.7)	153 (50.8)	109 (64.5)	
Past smoker	106 (22.6)	67 (22.3)	39 (23.1)	
Never smoked	101 (21.5)	80 (26.6)	21 (12.4)	
Ethnicity, white, n (%)	388 (82.6)	255 (84.7)	133 (78.7)	.102
Disease characteristics				
Hurley stage, n (%)				<.001
Mild (I)	159 (33.8)	117 (38.9)	42 (24.9)	
Moderate (II)	246 (52.3)	162 (53.8)	84 (49.7)	
Severe (III)	65 (13.8)	22 (7.3)	43 (25.4)	
HSS, median (range)	13 (0‐221)	12 (0–221)	16 (0‐180)	.017
Boils, median (range)	1 (0‐20)	1 (0‐20)	1 (0‐20)	.726
Patients with ≥1 boil, n (%)	324 (69.4)	213 (71.5)	111 (65.7)	.21
Blood profile, median (IQR)				
ALP [35‐105 U/L]	73 (59–89)	69 (56‐85)	81 (66‐96)	< .001
ALAT [10‐45 U/L] [10‐70 U/L]	21 (16‐29)	19 (15‐26)	27 (19‐38)	< .001
CRP [<10 mg/L]	3 (1‐8)	3 (1‐7)	4 (2‐8.5)	.018
ESR [2‐42 mm] [2‐30 mm]	10 (5‐18.75)	10.5 (5‐18)	9 (5‐21.25)	.137
Leukocytes [3.5‐8.8 × 10^9^/L]	8.4 (6.8‐10.4)	8.2 (6.7‐10)	8.85 (7‐10.8)	.019
Neutrophils [2‐8.8 × 10^9^/L]	5.1 (3.9‐6.5)	4.9 (3.9‐6.3)	5.5 (4‐7.3)	.032
NLR	2.24 (1.68‐3)	2.19 (1.7‐2.9)	2.39 (1.66‐3.12)	.011
Glucose [4.2‐6.3 mmol/L]	5.6 (5.2‐6.2)	5.5 (5.1‐6.1)	5.7 (5.4‐6.5)	.019
HDL [>1.2 mmol/L] [>1 mmol/L]	1.27 (1.03‐1.6)	1.37 (1.13‐1.64)	1.15 (0.9‐1.42)	.001
LDL [<3 mmol/L]	2.6 (2‐3.2)	2.6 (2‐3.2)	2.5 (2‐3.1)	.28
Triglycerides [0.45‐2.6 mmol/L]	1.31 (0.89‐1.96)	1.29 (0.85‐1.88)	1.44 (0.92‐2.22)	.028
Cholesterol [<5 mmol/L]	4.6 (4‐5.2)	4.7 (4‐5.35)	4.5 (3.9‐5.2)	.03
MetS criteria, n (%)				
Hypertension	339 (72.1)	206 (68.4)	133 (78.7)	.017
Obesity	175 (38.0)	122 (41.5)	53 (31.7)	.038
Diabetes	45 (9.6)	21 (7)	24 (14.2)	.011
Elevated triglycerides	151 (32.1)	86 (28.6)	65 (38.5)	.028
Dyslipidemia (HDL)	177 (39.9)	123 (43.2)	54 (34)	.058
>3 MetS criteria^a^	144 (33.0)	90 (32.1)	54 (34.4)	.631

Abbreviations: ALAT, alanine aminotransferase; ALP, alkaline phosphatase; CRP, C‐reactive protein; ESR, erythrocyte sedimentation rate; HDL, high‐density lipoprotein; HSS, hidradenitis suppurativa score; IQR, interquartile range; LDL, low‐density lipoprotein; MetS, metabolic syndrome; NLR, neutrophils‐lymphocytes ratio. Boils are number of boils in the past month.

*Note*: Obesity = BMI > 30, non‐fasting glucose above 11.1 mmol/L, Systole > 130 mmHg, Diastole > 85 mmHg.

^a^ 21 female and 12 male patients were excluded because of one or more criteria missing.

The prevalence and risk factors of elevated liver enzymes, disease severity, and MetS were explored by descriptive statistics. Cross tabulation was used for chi‐square and risk estimation. One‐way analysis of variance was used for analyzing the number of MetS criteria, alkaline phosphatase (ALP), and alanine aminotransferase (ALAT) levels between Hurley stages. Association and mean difference (MD) between age and presence of MetS and distribution of continuous variables between sexes were analyzed with independent *t* test. All tests were considered statistically significant at a *P*‐value of <.05 and 95% confidence interval.

## 3 RESULTS

The hepatic status was investigated by analyzing ALP and ALAT. The median ALP and ALAT levels were 73 U/L (IQR 59‐89) and 21 U/L (IQR 16‐29), respectively. Unadjusted analysis showed that ALP levels correlated with markers of systemic inflammation in females; C‐reactive protein (CRP), erythrocyte sedimentation rate (ESR), leukocytes, neutrophils, and neutrophil‐lymphocyte ratio (NLR), whereas among males, ALP levels only correlated significantly with CRP. In males, ALAT levels were negatively correlated with CRP, ESR, leukocytes, neutrophils, and NLR, whereas in females, ALAT levels were positively correlated with CRP and ESR (Table 2). When adjusting for the most common risk factors for nonalcoholic fatty liver disease (age, smoking, BMI, diabetes, and triglyceride levels^4^), ALP levels associated significantly with CRP and ESR in both sexes (*r* = .59, *P* < .001 in females and *r* = .40, *P* = .034 in males) and (*r* = .58, *P* < .001 in females and *r* = .47, *P* = .033 in males), respectively, and leukocytes, neutrophils, and NLR in females only (*r* = .52, *P* = .005; *r* = .47, *P* = .001; and *r* = .24, *P* = .018). In adjusted analysis, ALAT correlated with CRP in males (*r* = .42, *P* = .001), and neutrophils (*r* = .45, *P* = .022) and NLR (*r* = .22, *P* = .006) in females. There were no significant correlations among ALAT, ESR, and leukocytes in either sex.

**TABLE 2:**
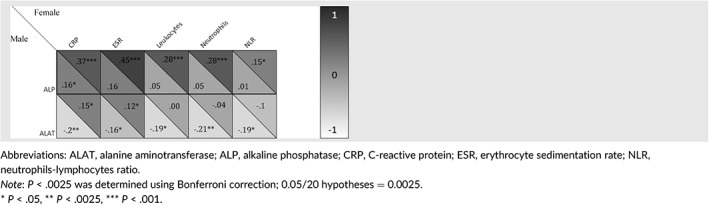
Correlogram of alkaline phosphatase, alanine aminotransferase and markers of inflammation

A total of 39 patients received tetra‐, lyme‐, or doxycycline, and nine patients received rifampicin at the time of visit. Although long‐term treatment with antibiotics is known to possibly affect hepatic function, we found no significant increase in ALP or ALAT levels (MD 7.2, *P* = .09) and (MD 0.4, *P* = .87), respectively, compared with those not receiving antibiotics.

Mean levels of ALP and ALAT in patients with Hurley stages I, II, and III were 69, 79, and 94 U/L, *P* < .001, and 23, 27, and 23 U/L, *P* = .022, respectively. In unadjusted analysis, only ALP was correlated with hidradenitis suppurativa score (HSS); in females (*r* = .28, *P* < .001) and males (*r* = .24, *P* = .002), even after adjustment (*r* = .31, *P* = .008 and *r* = .39, *P* < .001 in females and males, respectively). Only ALP levels correlated with the number of boils in the last month in females (*r* = .13, *P* = .024) but not in males (*r* = .11, *P* = .162), and for females even after adjustment (*r* = .30, *P* = .006).

**FIGURE 1:**
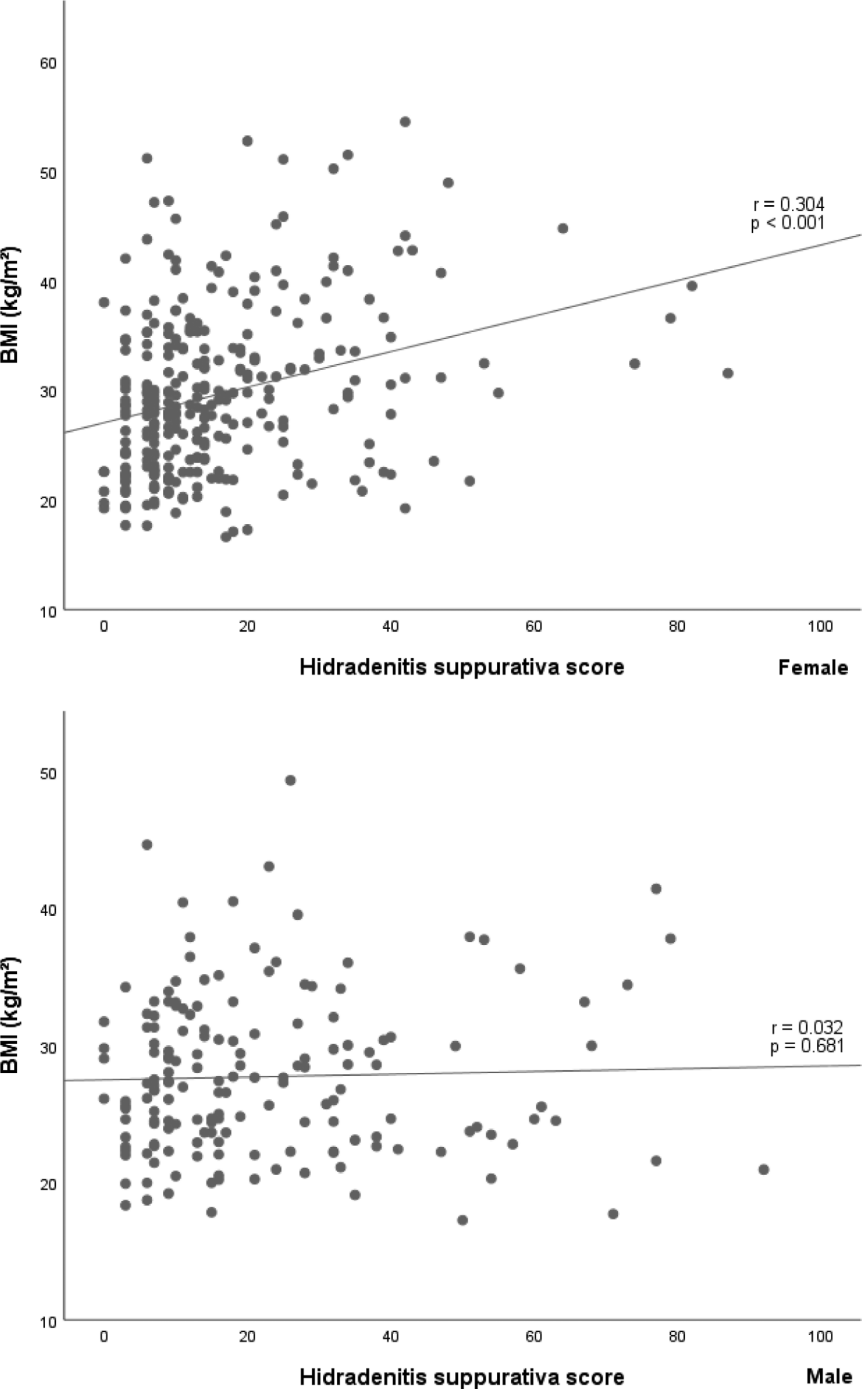
Scattergram of body mass index vs disease severity measured by hidradenitis suppurativa score

The prevalence of MetS (defined by the presence of at least three criteria, Table 1) was 32.1% in females and 34.4% in males. We found no significant difference in the distribution of MetS between Hurley stages (*P* = .468), or when split by sex, with a prevalence for females of 24.8%, 36.4%, and 40.0% (*P* = .103) and 41.5%, 32.9%, and 30.0% for males (*P* = .515) in Hurley stages I, II, and III, respectively. However, there was a significant difference in the number of criteria met (MetS score, 1‐5) between the Hurley stages in females (*P* = .005), but not in males (*P* = .79) where the association between MetS score and Hurley stage was modified by sex (*P* = .039). MetS score was correlated with HSS and disease duration in females (*r* = .25, *P* < .001) and (*r* = .24, *P* < .001), but not in males (*r* = .15, *P* = .063) and (*r* = .28, *P* = .12), respectively. HSS correlated with BMI in female patients (*r* = .304, *P* < .001) but not in males (*r* = .032, *P* = .681) (Figure 1). In addition, the presence of MetS associated with higher age in females (*P* < .001), but not in males (*P* = .65).

ALP levels were significantly higher in female patients with MetS (mean without MetS; 70.58 [SD 25.79], with MetS; 80.66 [SD 24.95], *P* = .002) as were ALAT levels (24.26 [SD 13.05]; 25.25 [SD 15.98], *P* = .028), but not for male patients. ALP levels correlated significantly with number of MetS criteria in females (*r* = .20, *P* = .001) but not in males (*r* = −.27, *P* = .739), whereas ALAT was significantly correlated with MetS score for females (*r* = .15, *P* = .014) and males (*r* = .17, *P* = .03).

A total of 12.9% had ALP above the reference level of 105 U/L and 5.3% had ALAT above the reference level of 70 U/L for males and 45 U/L for females.^5^ Males more often had elevated ALP levels compared with females (17.6% vs 10.3%), OR = 1.86 (CI 1.07‐3.22), *P* = .026, whereas there was no difference in elevated ALAT levels between the sexes (6.5% in females vs 3% in males), *P* = .11. In the subgroup of patients with abnormally high ALP levels, inflammatory markers of CRP, ESR, leukocytes, neutrophils, and NLR were significantly higher compared with those below the reference level, with an MD of 8.4, 14.2, 1.4, 1.2, and 0.5, respectively (all *P* < .003). Moreover, this subgroup had significantly higher disease severity, with an MD in HSS of 14.3 (*P* < .001) including a larger fraction of patients with Hurley III (32.2% vs 11.6%, *P* < .001), and presented with a larger mean MetS score (2.65 vs 1.93, *P* = .012) compared with those below the reference level.

## 4 DISCUSSION

A large percentage of our female patients were obese, which associate with an abnormal cytokine production,^6^ and thereby a chronic inflammatory response. Fat accumulation in the liver also stimulates hepatic cytokine production, which may be why we, especially in females, found a strong correlation of ALP with inflammatory biomarkers ESR and CRP. When combined with higher disease severity of HS, the inflammatory load increases, and thus increasing the risk of MetS, which may explain why the correlation between ALP and the presence of MetS was only significant for females.

Previous studies have shown that risk of type 2 diabetes and more advanced stages of liver disease increases when the number of MetS criteria increases,^7^, ^8^ supporting the importance of not only focusing on the presence of MetS, but also the number of criteria met.

Even though we found no significant difference in the presence of MetS between females and males, the number of criteria was dependent on HS severity and female sex. Based on this association, including the correlation between ALP levels and the presence of MetS, we propose the use of ALP as a predictor for MetS in patients with HS. This test allows the clinician to quickly determine whether their patients with abnormal liver results possibly are in high risk for MetS and refer to appropriate care.

## AUTHOR CONTRIBUTIONS

Conceptualization: Simon Francis Thomsen

Investigation: Jesper Grønlund Holm, Astrid‐Helene Ravn Jørgensen, Yiqiu Yao, Hans Christian Ring, Simon Francis Thomsen

Formal analysis: Valdemar Wendelboe Nielsen, Simon Francis Thomsen

Writing ‐ original draft: Valdemar Wendelboe Nielsen

Writing ‐ review & editing: Valdemar Wendelboe Nielsen, Jesper Grønlund Holm, Astrid‐Helene Ravn Jørgensen, Yiqiu Yao, Hans Christian Ring, Simon Francis Thomsen.

## TRANSPARENCY STATEMENT

The corresponding author confirms that the manuscript is an honest, accurate, and transparent account of the study being reported; that no important aspects of the study have been omitted; and that any discrepancies from the study as planned have been explained.

## Data Availability

Data sharing is not applicable to this article, due to legislation by the Danish Data Protection Agency.
